# The Voice of Physicians: The Essential Role of Public Speaking in Medical Training

**DOI:** 10.1007/s40670-025-02419-3

**Published:** 2025-05-29

**Authors:** Emily Kwon, Rijul Asri, Jeremy Grachan, Christin Traba

**Affiliations:** 1 Office of Education, Rutgers New Jersey Medical School, Newark, NJ USA; 2 Department of Psychiatry, Rutgers New Jersey Medical School, Newark, NJ USA; 3 Department of Pediatrics, Rutgers New Jersey Medical School, Newark, NJ USA; 4 Department of Medicine, Rutgers New Jersey Medical School, Newark, NJ USA

**Keywords:** Undergraduate medical education, Public speaking, Virtual presentations, In-person presentations

## Abstract

Public speaking skills are essential for career advancement and success in medicine. This needs assessment evaluated the public speaking challenges faced by medical students and faculty, identifying areas where targeted training can enhance communication skills crucial for medical practice. Size and demographics (e.g., students versus attendings) of the audience population affect public speaking confidence, particularly for students. Both groups prefer practical, interactive learning methods, like workshops and peer practice. Medical schools can integrate training and practice opportunities into existing curricula, ensuring that students still receive essential communication skills development within their current programs.

**Supplementary Information:**

The online version contains supplementary material available at 10.1007/s40670-025-02419-3.

## Background

In 1995, the addition of instruction and evaluation of communication skills was adopted into the Standards for Accreditation of Medical Education Programs Leading to the M.D. Degree [1]. Some schools have implemented experimental pre-clerkship communications curricula to improve physicians’ interactions with patients and families, focusing on empathy, compassion, and conveying clinical information [2, 3]. Beyond patient interactions, physicians give presentations across venues, including grand rounds, national presentations, news media, and social media [4]. To date, however, there has been limited guidance on the specifics of how the education of communication skills, especially skills beyond interactions with patients and families, should be implemented [5]. Conveying information effectively to the target audience is equally important to understanding the content, an issue that affects other graduate fields as well [6]. Further, the Association of American Medical Colleges (AAMC) highlights the importance of presenting scholarly work at national meetings to advance health care and to also expand one’s professional network [5]. Medical schools have also incorporated interview training programs to improve student confidence in residency interviews as interpersonal skills and professionalism are important factors program directors use in ranking applicants [7].

Beyond the training setting, many studies have identified effective self-presentation, communication, and public speaking as important “soft skills” for professional success and career advancement [8, 9]. The AAMC, in collaboration with the Accreditation Council for Graduate Medical Education (ACGME), has launched initiatives to establish foundational competencies for medical schools, including interpersonal and communication skills [10]. These competencies align with the ACGME competencies, underscoring that integrating public speaking into medical training supports a continuum of professional development from undergraduate to graduate medical education and beyond to clinical practice [10].

Despite the recognized importance, public speaking is often underrepresented in medical curricula. Prior needs assessments have addressed the need for public speaking training in undergraduate medical education (UME) internationally; however, there are few studies focusing on this need within the USA [11–14]. A recent scoping review also indicates that communication skills training remains poorly represented and inconsistently implemented in medical school curricula globally [11]. This underrepresentation stems from the densely packed medical curriculum, which prioritizes essential clinical knowledge and skills, leaving limited space for additional topics, as well as limited institutional support, and at times undervalued communication skills relative to biomedical content [12, 15, 16]. Bandura’s Self-Efficacy Theory posits that individuals’ belief in their ability to perform a specific task strongly influences their motivation, persistence, and performance [17]. Faculty, having accumulated more experience with academic presentations and clinical communication, are likely to have developed greater self-efficacy in public speaking. In contrast, students with fewer structured opportunities to speak in professional settings may exhibit lower confidence and increased performance anxiety.

This study sought to identify the attitudes toward the importance, relevance, and practice of public speaking of medical students and faculty at Rutgers New Jersey Medical School (NJMS) through a cross-sectional needs assessment. While it is hypothesized that faculty will be more confident in public speaking, identifying specific areas of need from both student and faculty perspectives can help establish a foundation for structured, contextually relevant curricular interventions that can be piloted and scaled to align with the AAMC and ACGME competencies [10].

## Activity

The needs assessment survey was distributed to 681 NJMS medical students (year 1, 176; year 2, 174; year 3, 175; year 4, 156) and 575 faculty members. The 24-question survey included multiple-choice, Likert scale, and open-ended questions (Supplementary Material 1). The survey was developed from online Google searches of previously published public speaking surveys [18, 19]. Prior to dissemination, the survey was piloted and revised based on feedback from four medical students and three faculty.

The survey was administered through Rutgers University Qualtrics and disseminated via email listservs with electronic informed consent (Rutgers, The State University of New Jersey Institutional Review Board Pro2024000267). All data was coded and analyzed with IBM Statistical Package for the Social Sciences Version 29. Only responses with at least 90% completion were included in the analysis. The number of subjects for each analysis and the statistical test utilized is noted for each variable explored in the “Results” section. The open-ended response data were independently reviewed by two team members, who each identified key themes using thematic analysis. Discrepancies in themes were discussed (less than 5% of the time) and consensus obtained through reevaluating in collaborative meetings to incorporate themes into a single, cohesive list.

The modest sample size (94 responses; 7.48% response rate) introduces the potential for nonresponse bias, and the perspectives collected may not fully represent those of the larger NJMS community. As such, findings should be considered preliminary, and interpreted with caution, acknowledging the limited generalizability of the results.

## Results and Discussion

A total of 51 students and 43 faculty members completed the survey. All students were between the ages of 18 and 35, whereas most of the faculty were over 35 years old. An independent samples *t*-test was performed to compare opinions of public speaking between students and faculty (Table 1). Both students and faculty rated the importance of public speaking highly and felt motivated to improve their public speaking skills. However, faculty exhibited significantly more confidence, better self-perceived skills, and greater frequency of both in-person and virtual public speaking activities. These findings are in line with Kolb’s theory [20]. Faculty, through their professional roles, have cycled through learning stages more often regarding public speaking, leading to higher confidence. Students, with fewer experiences, may be stuck in a cycle of anxiety and avoidance.

**Table 1:** Comparison of Likert scales between faculty and students regarding opinions of public speaking

Survey question	Student (*n* = 51)		Faculty (*n* = 43)		*p*-value^b^
	Mean	SD^a^	Mean	SD^a^	
How often do you currently engage in public speaking activities (e.g., lectures, research presentations, interviews) in person?^c^	2.23	0.709	3.46	0.959	*p* < 0.001
How often do you currently engage in public speaking activities (e.g., lectures, research presentations, interviews) virtually?^c^	2.09	0.412	3.11	0.980	*p* < 0.001
On average, how confident do you currently feel when speaking in public?^d^	2.66	0.993	3.65	0.948	*p* < 0.001
On average, how good of a public speaker do you think you are?^d^	3.13	0.775	3.76	0.811	*p* < 0.001
How important do you think public speaking skills are for your professional success?^e^	4.39	0.532	4.53	0.549	*p* = 0.205
How motivated are you to improve your public speaking?^e^	3.76	0.885	3.53	1.31	*p* = 0.333

^a^Standard deviation

^b^Independent samples *t*-test

^c^Rated on a 5-point scale (1 = never, 5 = very frequently, more than 2 times per week)

^d^Rated on a 5-point scale (1, not confident at all; 5, extremely confident)

^e^Rated on a 5-point scale (1, not important at all; 5, extremely important)

Only 33% of students and 47% of faculty had formal public speaking training, which included extracurricular activities, formal public speaking education, and integration within school curricula or jobs.

Respondents’ explanations of their self-rated public speaking proficiency were analyzed, and six key themes were identified: confidence and anxiety; preparation and practice; experience and skill; delivery and engagement; feedback and evaluation; and context and topic dependence (Table 2). Confidence and anxiety strongly influenced perceptions of public speaking skill level, corroborating findings from Rossi-Barbosa et al. [21]. Respondents frequently emphasized the importance of rehearsal and thorough preparation, echoing research highlighting the link between practice and self-perceived competence [22]. These findings highlight the multifaceted nature of public speaking and the wide range of factors influencing individual perceptions and performance.

**Table 2:** Open-ended responses to “How good of a public speaker do you think you are?”

Theme	Sample quotations
Confidence and anxiety	“Terrified of public speaking…”; “I feel very confident…”; “I get performance anxiety…”
Preparation and practice	“Usually, I have to practice many times…”; “If I have to present without practice, then the presentation does not go well…”
Experience and skill	“Not enough experience…”; “I think that I am naturally comfortable…”; “I have a lot of experience and have learned my strengths and weaknesses…”
Delivery and engagement	“Once I get in the rhythm I feel better…”; “I think that I'm an energetic and reasonably engaging public speaker…”
Feedback and evaluation	“Consistently get the highest student evaluations…”; “I have repeatedly received excellent feedback…”; “student evaluations always rank 4.5 or above…”
Context and topic dependence	“It depends on the topic…”; “Sometimes I present nationally”

**Fig. 1:**
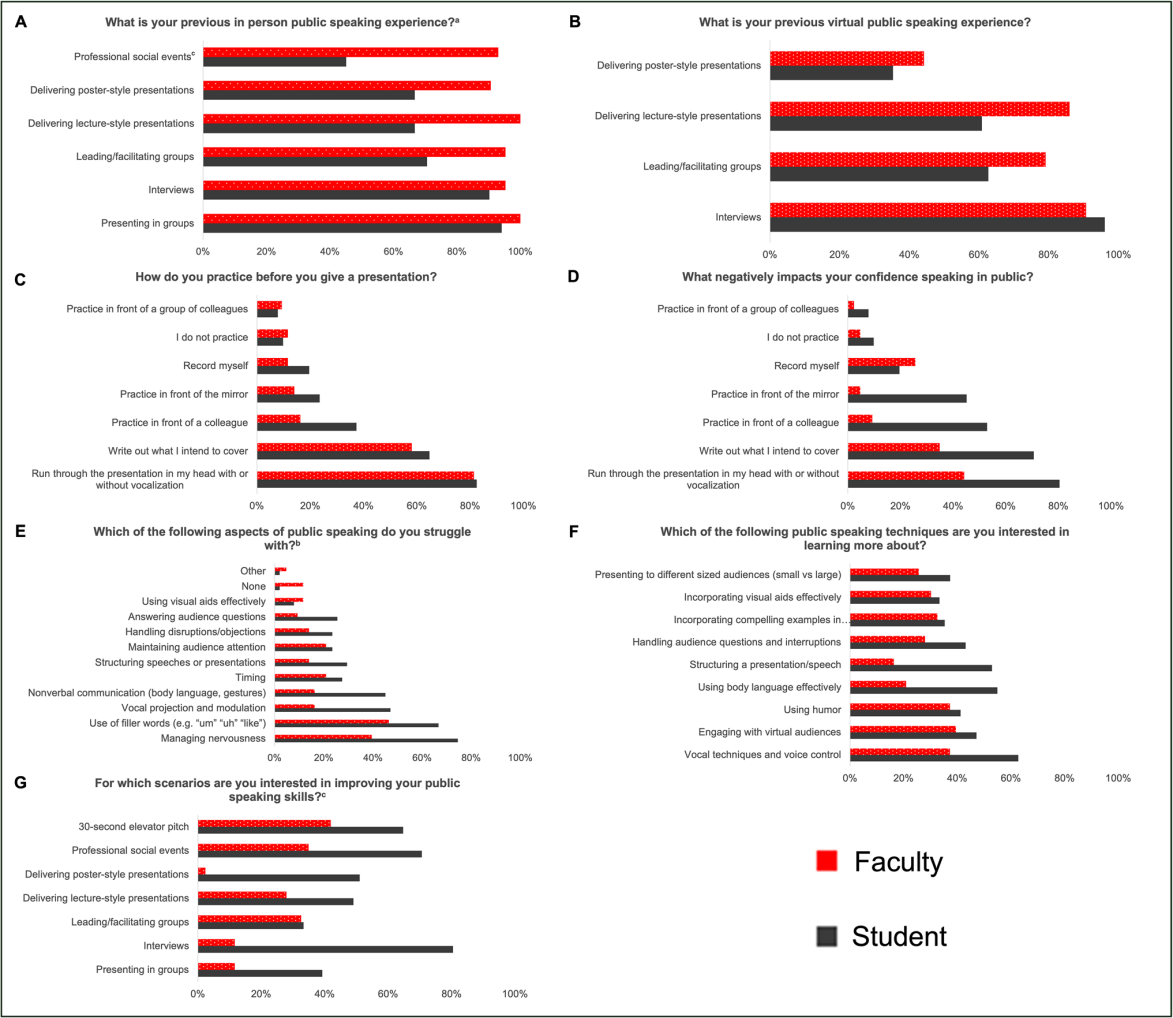
Comparison of proportion of student and faculty response regarding public speaking experiences, practice methods, and factors impacting speaking. Respondents (51 students; 43 faculty) could select multiple responses. ^a^One person reported they had engaged in public speaking for radio and TV. ^b^Three respondents referred to stuttering, facial expressions, improvising, or imposter syndrome as aspects of public speaking they struggled with. ^c^Professional social events included residency interview socials and conference events

A comparison of student and faculty experiences, practice methods, and areas of interest for skill improvement is presented in Fig. 1. A large audience, as well as audience professional level, negatively impacted students’ (70.6% and 80.4%, respectively) and faculty’s (34.9% and 44.2%, respectively) speaking confidence (Fig. 1D). Managing nervousness and avoiding filler words were the most common struggles for students (74.5% and 66.7%, respectively) and faculty (39.5% and 46.5%, respectively) (Fig. 1E). Both groups wanted to improve vocal techniques, engage virtual audiences, and master body language and speech structuring (Fig. 1F).

Students were particularly interested in improving skills such as interview techniques (80.4%), elevator pitches (64.7%), group presentations (39.2%), and poster presentations (51%)—key activities they engage in during medical school to prepare for residency (Fig. 1G) [6]. This interest in skill enhancement is corroborated by others, who found that students expressed a desire for public speaking classes in their curriculum to improve their skills [23]. On the other hand, faculty, having previously experienced these earlier skills, focus more on presenting to large audiences and delivering lectures, which aligns with their professional responsibilities (Fig. 1F, G). Both groups preferred practical, interactive learning methods, such as workshops and peer practice, emphasizing the opportunity to offer structured public speaking training as an option for those who wish to further develop their skills. The demographics and size of the audience affected confidence, particularly for students, suggesting a need for targeted training to help build confidence in diverse speaking situations.

The rise of virtual public speaking through social media platforms, online lectures, political campaigns, residency interviews, and grand rounds is reshaping the landscape [24]. It is crucial for medical education to address this shift and equip students and faculty with the skills to effectively engage in both traditional and virtual public speaking formats. This necessitates a focus on developing competencies in both modalities, ensuring that learners are prepared for the diverse communication demands of the modern medical field.

### Limitations and Future Directions

The single-center design and low response rate limit the generalizability of our findings. Additionally, the reliance on self-reported data may introduce bias, as participants’ perceptions of their own public speaking skills and experiences may not always align with objective measures. Despite these limitations, the study provides preliminary insights into the public speaking needs of medical students and faculty, informing future curricular development efforts.

Future research should address these limitations by employing larger, multi-institutional samples and incorporating objective measures of public speaking competence. Furthermore, future work should consider intervention-based assessments to evaluate the effectiveness of different pedagogical approaches. For example, the impact of incorporating public speaking training as dedicated modules, longitudinal elective courses, or simulation-based workshops could be rigorously assessed using pre- and post-intervention measures.

At NJMS, active discussions are underway to integrate public speaking opportunities throughout the pre-clerkship and clinical years, carefully considering the specific needs of each stage. For instance, a dedicated pre-clerkship communication skills module could concentrate on foundational skills utilizing standardized patients. During the third and fourth years, workshops could address clinical presentation delivery and poster presentations. As students transition to the fourth year, workshops on interview skills, potentially leveraging AI resources for personalized feedback, could be valuable [25]. This approach aligns with the transactional model of communication, which views communication as a dynamic and reciprocal process between speaker and audience. This model underscores the importance of experiential learning and feedback loops—key components in cultivating both competence and confidence in speaking [26]. Future research could then evaluate the relative impact and optimal integration strategies of these diverse approaches.

## Conclusions

The findings of this study advocate for the strategic inclusion of public speaking development within medical training. By directly addressing the interpersonal and communication skills that the AAMC and ACGME have established as core competencies, this curricular integration will better equip medical professionals for success in all facets of their careers. From residency interviews and academic presentations to advocacy using social media, confident and effective public speaking will enable them to fulfill these essential professional development goals.

## Supplementary Information

Below is the link to the electronic supplementary material.

Supplementary file1 (DOCX 35 KB)

## Data Availability

Raw data that support the findings of this study are available from the corresponding author, upon reasonable request.
