# Supplementary material for: The Voice of Physicians: The Essential Role of Public Speaking in Medical Training
Source: Med Sci Educ. 2025 May 29;35(4):1887–92. doi: 10.1007/s40670-025-02419-3 (PMC12532966; doi:10.1007/s40670-025-02419-3)
Supplement: Supplementary file 1 — Supplementary file1 (DOCX 35 KB) [file 40670_2025_2419_MOESM1_ESM.docx]

Supplementary Material 1

**Article Title:** *The Voice of Physicians: The Essential Role of Public Speaking in Medical Training*

**Journal name:** Medical Science Educator

**Author names:** Emily Kwon, BA, Corresponding Author; Rijul Asri, MD; Jeremy J. Grachan, Ph.D; Christin Traba, MD, MPH

**Affiliation of corresponding author:** Office of Education, Rutgers New Jersey Medical School, Newark, NJ, USA

**Email of corresponding author:** [esk104@njms.rutgers.edu](mailto:esk104@njms.rutgers.edu)

Needs Assessment

1. Which of the following best describes your current professional stage?

- Medical Student
- Attending Faculty
- Teaching Faculty

1. (Medical Student Only) What year are you?

- M1
- M2
- M3
- M4
- Other

1. How old are you?

- Under 18
- 18-25
- 26-35
- 36-45
- 46-55
- 56-65
- 65+

1. Which degree(s) do you have? Select all that apply.

- MBA
- MPP
- MPH
- MHA
- Other Master’s Degree
- MD
- DO
- PhD
- JD
- PharmD
- DMD
- DPT
- Other

1. (Medical Student Only) What specialty are you interested in pursuing?
2. (Attending/Teaching) What is your specialty?
3. (Attending/Teaching) What is your primary work or practice setting (select all that apply)

- private practice
- medical school
- teaching hospital
- Community hospital
- Non-clinical non-teaching role
- Other (elaborate)

**Public speaking** is the process of communicating information to an audience. It can range from interviewing for residency, leading small group sessions, speaking to news media, to lecturing to audiences of hundreds.

1. Which of the following public speaking activities have you engaged before **in person**? Select all that apply.

- Interviews
- Professional social events (e.g. residency interview socials, conference events)
- Presenting in groups
- Leading/facilitating groups
- Delivering poster-style presentations
- Delivering lecture-style presentations
- Other (please specify)

1. How often do you currently engage in public speaking activities (e.g., lectures, research presentations, interviews) **in person**?

- Never
- Rarely (less than once per month)
- Occasionally (1-3 times per month)
- Frequently (1-2 times per week)
- Very frequently (more than 2 times per week)

1. Which of the following public speaking activities have you engaged before **virtually**? Select all that apply.

- Interviews
- Professional social events (e.g. residency interview socials, conference events)
- Presenting in groups
- Leading/facilitating groups
- Delivering poster-style presentations
- Delivering lecture-style presentations
- Other (please specify)

1. How often do you currently engage in public speaking activities (e.g., lectures, research presentations, interviews) **virtually**?

- Never
- Rarely (less than once per month)
- Occasionally (1-3 times per month)
- Frequently (1-2 times per week)
- Very frequently (more than 2 times per week)

1. Have you had any training, classes, or workshops that provided guidance or instruction on presentation or public speaking?

- Yes, please explain
- No

1. How do you practice before you give a presentation? Select all that apply.

- I do not practice
- Run through the presentation in my head with minimal vocalization
- Practice in front of the mirror
- Practice in front of a colleague
- Practice in front of a group of colleagues
- Write out what I intend to cover
- Record myself
- Other (please specify)

1. On average, how confident do you currently feel when speaking in public?

- 1 – Not confident at all, 5 – Extremely confident

1. On average, how good of a public speaker do you think you are?

- 1 – Not confident at all, 5 – Extremely confident

1. Please explain your rating on the previous question.
2. Which of the following things negatively impact your confidence speaking in public? (select all that apply)

- Large audience size
- Small audience size
- Audience population (students, attendings, colleagues)
- In person presentations
- Virtual presentations
- Solo presentations
- Group presentations
- Other (please specify)

1. What aspects of public speaking do you struggle with? Select all that apply.

- Managing nervousness
- Structuring speeches or presentations
- Maintaining audience attention
- Answering audience questions
- Handling disruptions/objections
- Use of filler words (e.g. “um” “uh” “like”)
- Using visual aids effectively
- Nonverbal communication (body language, gestures)
- Vocal projection and modulation
- Timing
- Other (please specify)
- None

1. What aspect of public speaking do you struggle with **the most**?

- Managing nervousness
- Structuring speeches or presentations
- Maintaining audience attention
- Answering audience questions
- Handling disruptions/objections
- Use of filler words (e.g. “um” “uh” “like”)
- Using visual aids effectively
- Nonverbal communication (body language, gestures)
- Vocal projection and modulation
- Timing
- Other (please specify)
- None

1. How important do you think public speaking skills are for your professional success?

- 1 – not important at all, 5 – extremely important

1. How motivated are you to improve your public speaking?

- 1 – not important at all, 5 – extremely important

1. Which of the following public speaking techniques would you be interested in learning more about? Select all that apply.

- Structuring a presentation/speech
- Incorporating compelling examples in presentations/speeches
- Using humor
- Incorporating visual aids effectively
- Using body language effectively
- Vocal techniques and voice control
- Handling audience questions and interruptions
- Engaging with virtual audiences
- Presenting to different sized audiences (small vs large)
- Other (please specify)

1. For which of the following scenarios are you interested in improving your public speaking skills? Select all that apply.

- 30-second elevator pitch
- Interviews
- Presenting in groups
- Leading/facilitating groups
- Delivering poster-style presentations
- Delivering lecture-style presentations
- Professional social events (e.g. residency interview socials, conference events)
- Other (please specify)

1. What are your preferred learning methods for improving public speaking skills? Select all that apply.

- In-person workshops or seminars
- Online course (synchronous)
- Online course (asynchronous)
- Online courses (combination of synchronous and asynchronous)
- Practice with peers
- Reading books or articles
- Watching TED talks or example speeches
- Recording and analyzing own speeches
- Other (please specify)
